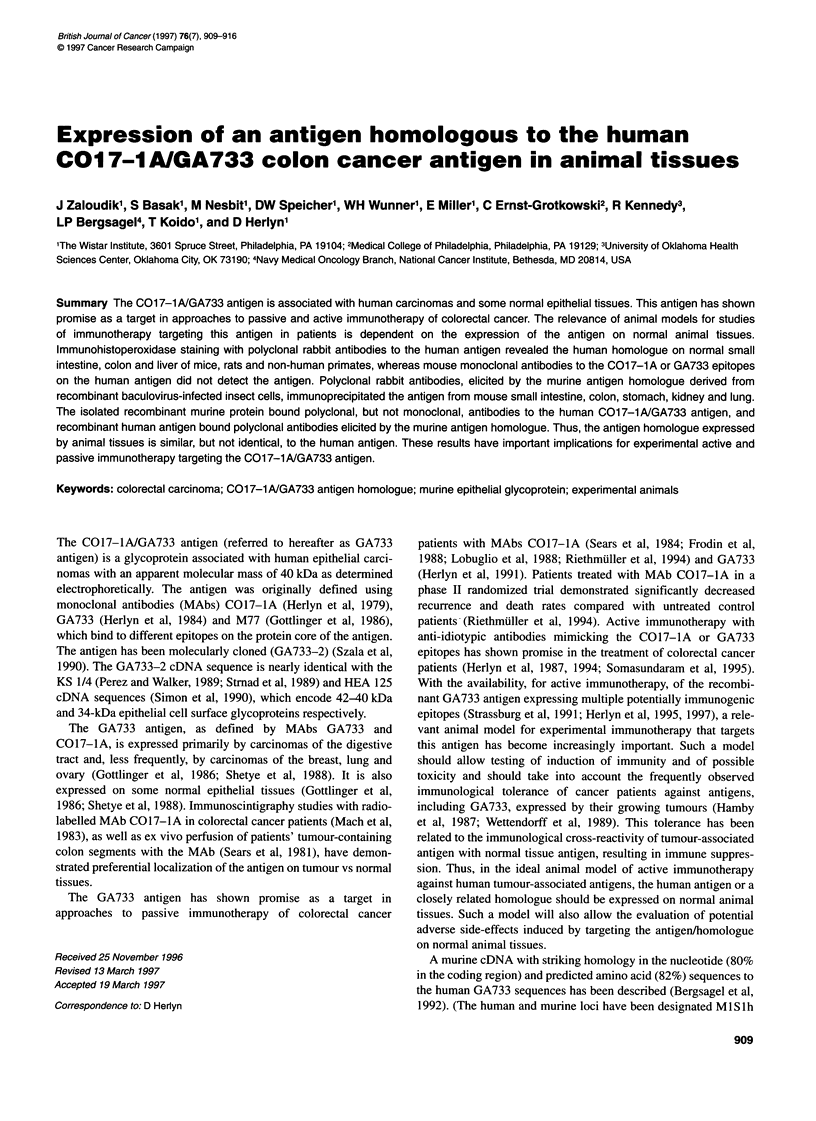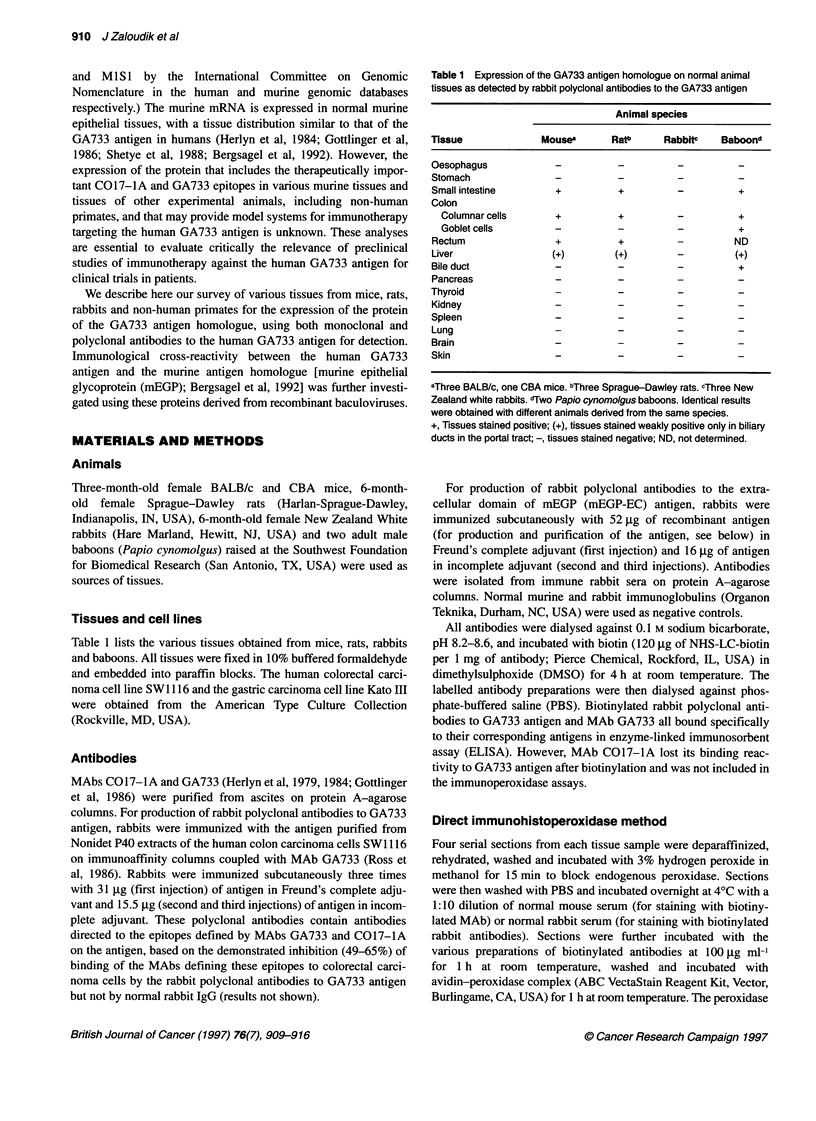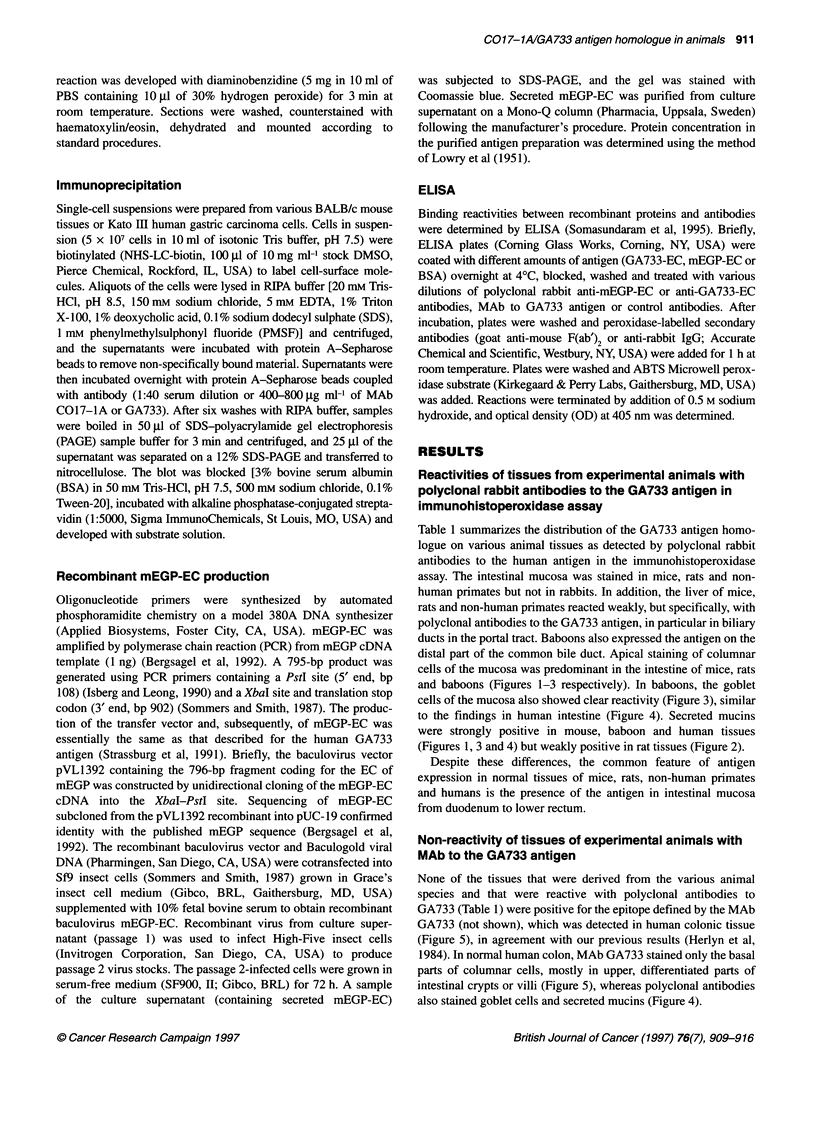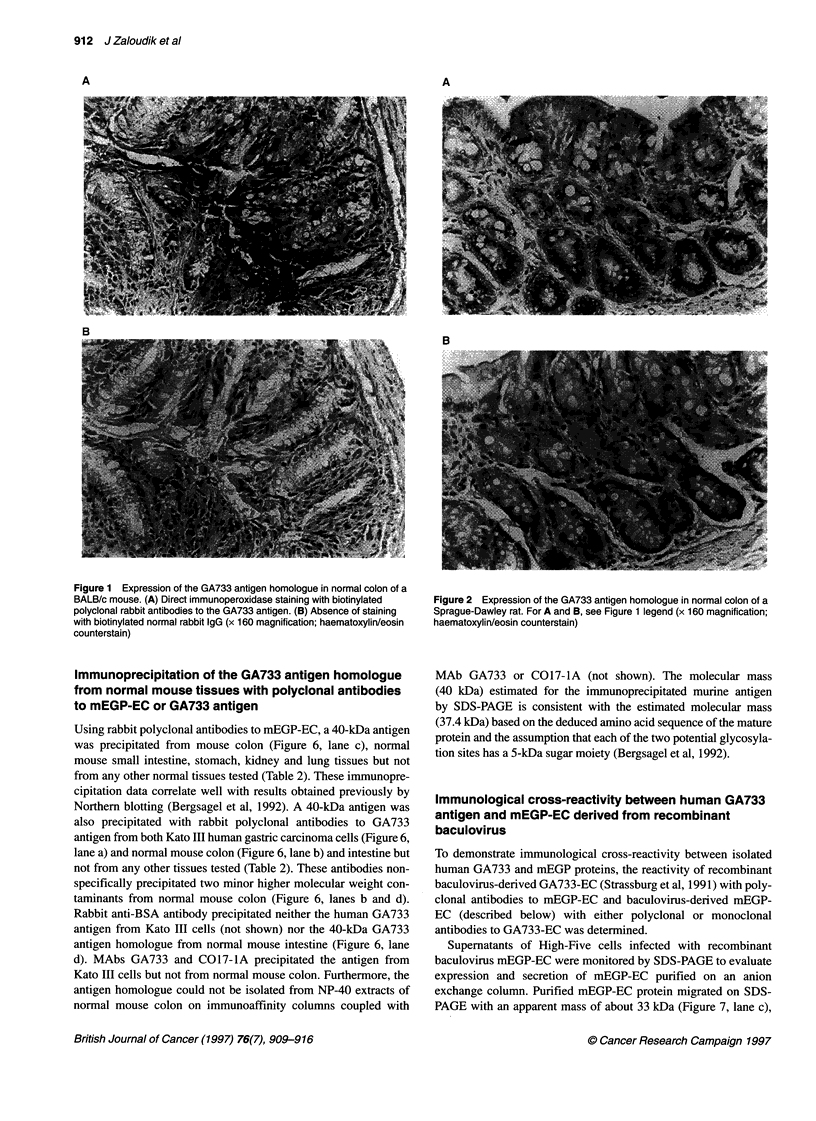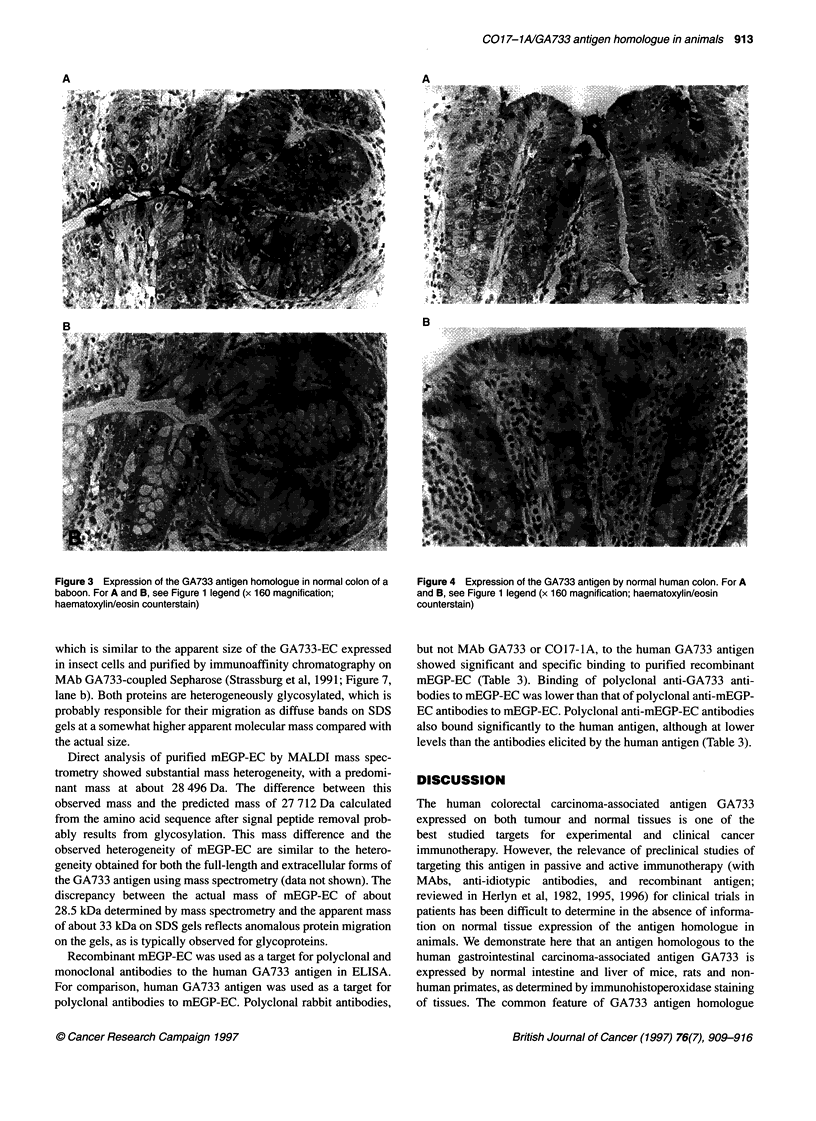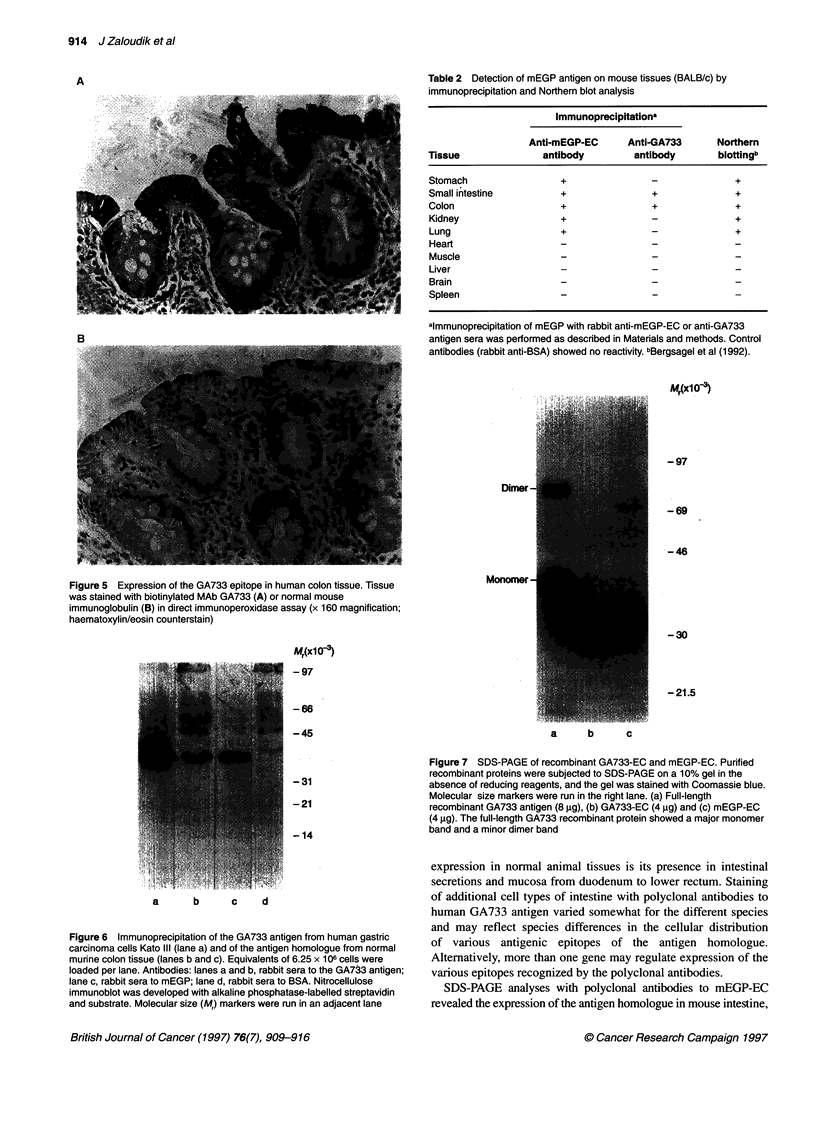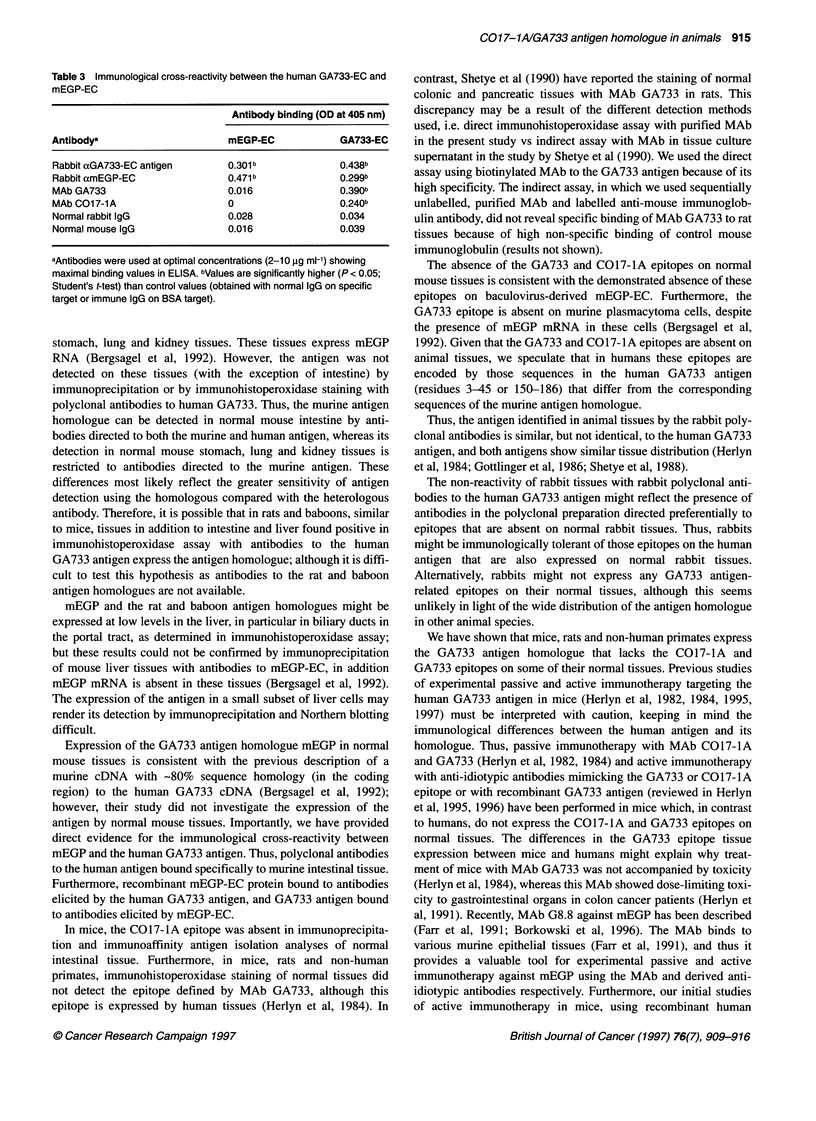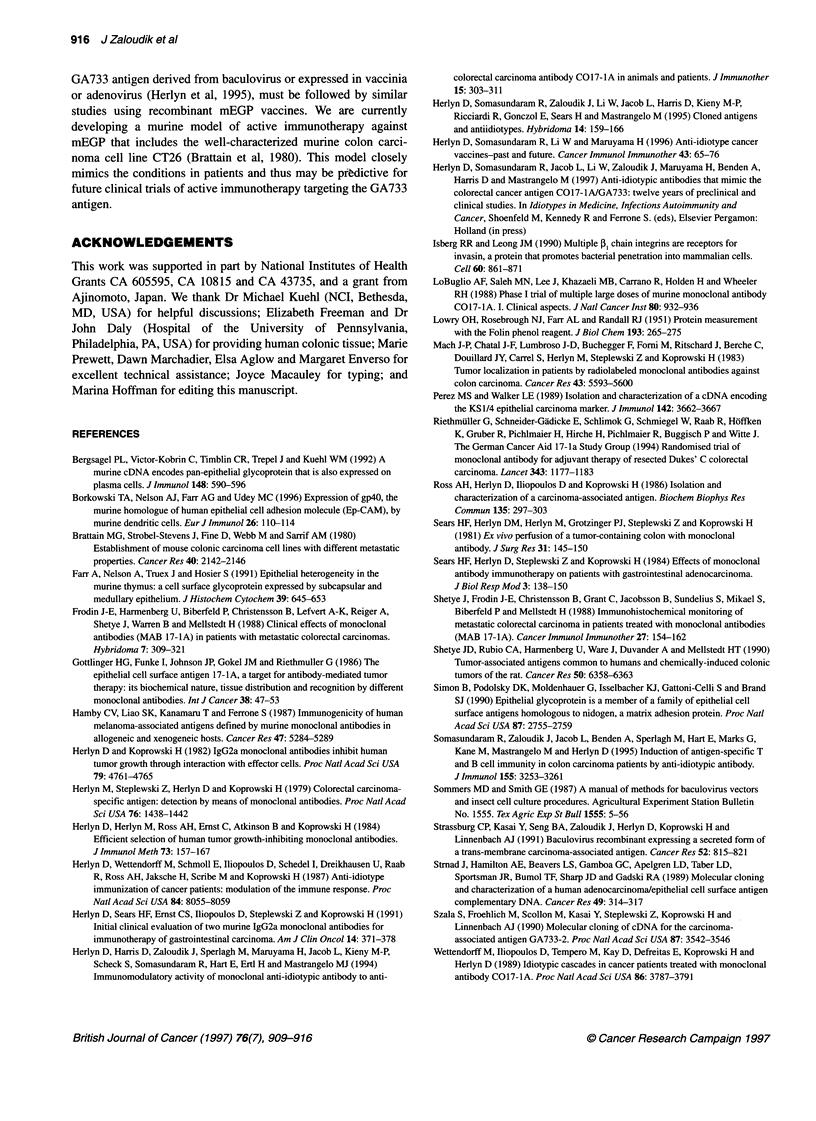# Expression of an antigen homologous to the human CO17-1A/GA733 colon cancer antigen in animal tissues.

**DOI:** 10.1038/bjc.1997.483

**Published:** 1997

**Authors:** J. Zaloudik, S. Basak, M. Nesbit, D. W. Speicher, W. H. Wunner, E. Miller, C. Ernst-Grotkowski, R. Kennedy, L. P. Bergsagel, T. Koido, D. Herlyn

**Affiliations:** The Wistar Institute, Philadelphia, PA 19104, USA.

## Abstract

**Images:**


British Journal of Cancer (1997) 76(7), 909–916
© 1997 Cancer Research Campaign

### Expression of an antigen homologous to the human CO17–1A/GA733 colon cancer antigen in animal tissues

**J Zaloudik[1], S Basak[1], M Nesbit[1], DW Speicher[1], WH Wunner[1], E Miller[1], C Ernst-Grotkowski[2], R Kennedy[3], LP Bergsagel[4], T Koido[1], and D Herlyn[1]**

[1]The Wistar Institute, 3601 Spruce Street, Philadelphia, PA 19104; [2]Medical College of Philadelphia, Philadelphia, PA 19129; [3]University of Oklahoma Health Sciences Center, Oklahoma City, OK 73190; [4]Navy Medical Oncology Branch, National Cancer Institute, Bethesda, MD 20814, USA

**Summary** The CO17–1A/GA733 antigen is associated with human carcinomas and some normal epithelial tissues. This antigen has shown promise as a target in approaches to passive and active immunotherapy of colorectal cancer. The relevance of animal models for studies of immunotherapy targeting this antigen in patients is dependent on the expression of the antigen on normal animal tissues. Immunohistoperoxidase staining with polyclonal rabbit antibodies to the human antigen revealed the human homologue on normal small intestine, colon and liver of mice, rats and non-human primates, whereas mouse monoclonal antibodies to the CO17–1A or GA733 epitopes on the human antigen did not detect the antigen. Polyclonal rabbit antibodies, elicited by the murine antigen homologue derived from recombinant baculovirus-infected insect cells, immunoprecipitated the antigen from mouse small intestine, colon, stomach, kidney and lung. The isolated recombinant murine protein bound polyclonal, but not monoclonal, antibodies to the human CO17–1A/GA733 antigen, and recombinant human antigen bound polyclonal antibodies elicited by the murine antigen homologue. Thus, the antigen homologue expressed by animal tissues is similar, but not identical, to the human antigen. These results have important implications for experimental active and passive immunotherapy targeting the CO17–1A/GA733 antigen.

**Keywords:** colorectal carcinoma; CO17–1A/GA733 antigen homologue; murine epithelial glycoprotein; experimental animals

The CO17–1A/GA733 antigen (referred to hereafter as GA733 antigen) is a glycoprotein associated with human epithelial carcinomas with an apparent molecular mass of 40 kDa as determined electrophoretically. The antigen was originally defined using monoclonal antibodies (MAbs) CO17–1A (Herlyn et al, 1979), GA733 (Herlyn et al, 1984) and M77 (Gottlinger et al, 1986), which bind to different epitopes on the protein core of the antigen. The antigen has been molecularly cloned (GA733–2) (Szala et al, 1990). The GA733–2 cDNA sequence is nearly identical with the KS 1/4 (Perez and Walker, 1989; Strnad et al, 1989) and HEA 125 cDNA sequences (Simon et al, 1990), which encode 42–40 kDa and 34-kDa epithelial cell surface glycoproteins respectively.

The GA733 antigen, as defined by MAbs GA733 and CO17–1A, is expressed primarily by carcinomas of the digestive tract and, less frequently, by carcinomas of the breast, lung and ovary (Gottlinger et al, 1986; Shetye et al, 1988). It is also expressed on some normal epithelial tissues (Gottlinger et al, 1986; Shetye et al, 1988). Immunoscintigraphy studies with radiolabelled MAb CO17–1A in colorectal cancer patients (Mach et al, 1983), as well as ex vivo perfusion of patients' tumour-containing colon segments with the MAb (Sears et al, 1981), have demonstrated preferential localization of the antigen on tumour vs normal tissues.

The GA733 antigen has shown promise as a target in approaches to passive immunotherapy of colorectal cancer patients with MAbs CO17–1A (Sears et al, 1984; Frodin et al, 1988; Lobuglio et al, 1988; Riethmüller et al, 1994) and GA733 (Herlyn et al, 1991). Patients treated with MAb CO17–1A in a phase II randomized trial demonstrated significantly decreased recurrence and death rates compared with untreated control patients (Riethmüller et al, 1994). Active immunotherapy with anti-idiotypic antibodies mimicking the CO17–1A or GA733 epitopes has shown promise in the treatment of colorectal cancer patients (Herlyn et al, 1987, 1994; Somasundaram et al, 1995). With the availability, for active immunotherapy, of the recombinant GA733 antigen expressing multiple potentially immunogenic epitopes (Strassburg et al, 1991; Herlyn et al, 1995, 1997), a relevant animal model for experimental immunotherapy that targets this antigen has become increasingly important. Such a model should allow testing of induction of immunity and of possible toxicity and should take into account the frequently observed immunological tolerance of cancer patients against antigens, including GA733, expressed by their growing tumours (Hamby et al, 1987; Wettendorff et al, 1989). This tolerance has been related to the immunological cross-reactivity of tumour-associated antigen with normal tissue antigen, resulting in immune suppression. Thus, in the ideal animal model of active immunotherapy against human tumour-associated antigens, the human antigen or a closely related homologue should be expressed on normal animal tissues. Such a model will also allow the evaluation of potential adverse side-effects induced by targeting the antigen/homologue on normal animal tissues.

A murine cDNA with striking homology in the nucleotide (80% in the coding region) and predicted amino acid (82%) sequences to the human GA733 sequences has been described (Bergsagel et al, 1992). (The human and murine loci have been designated M1S1h

*Received 25 November 1996*
*Revised 13 March 1997*
*Accepted 19 March 1997*

*Correspondence to:* D Herlyn and M1S1 by the International Committee on Genomic Nomenclature in the human and murine genomic databases respectively.) The murine mRNA is expressed in normal murine epithelial tissues, with a tissue distribution similar to that of the GA733 antigen in humans (Herlyn et al, 1984; Gottlinger et al, 1986; Shetye et al, 1988; Bergsagel et al, 1992). However, the expression of the protein that includes the therapeutically important CO17–1A and GA733 epitopes in various murine tissues and tissues of other experimental animals, including non-human primates, and that may provide model systems for immunotherapy targeting the human GA733 antigen is unknown. These analyses are essential to evaluate critically the relevance of preclinical studies of immunotherapy against the human GA733 antigen for clinical trials in patients.

We describe here our survey of various tissues from mice, rats, rabbits and non-human primates for the expression of the protein of the GA733 antigen homologue, using both monoclonal and polyclonal antibodies to the human GA733 antigen for detection. Immunological cross-reactivity between the human GA733 antigen and the murine antigen homologue [murine epithelial glycoprotein (mEGP); Bergsagel et al, 1992] was further investigated using these proteins derived from recombinant baculoviruses.

**Table 1** Expression of the GA733 antigen homologue on normal animal tissues as detected by rabbit polyclonal antibodies to the GA733 antigen

| | Animal species | | | |
|---|---|---|---|---|
| **Tissue** | **Mouse**[a] | **Rat**[b] | **Rabbit**[c] | **Baboon**[d] |
| Oesophagus | – | – | – | – |
| Stomach | – | – | – | – |
| Small intestine | + | + | – | + |
| Colon | | | | |
| Columnar cells | + | + | – | + |
| Goblet cells | – | – | – | + |
| Rectum | + | + | – | ND |
| Liver | (+) | (+) | – | (+) |
| Bile duct | – | – | – | + |
| Pancreas | – | – | – | – |
| Thyroid | – | – | – | – |
| Kidney | – | – | – | – |
| Spleen | – | – | – | – |
| Lung | – | – | – | – |
| Brain | – | – | – | – |
| Skin | – | – | – | – |

[a]Three BALB/c, one CBA mice. [b]Three Sprague–Dawley rats. [c]Three New Zealand white rabbits. [d]Two *Papio cynomolgus* baboons. Identical results were obtained with different animals derived from the same species.
+, Tissues stained positive; (+), tissues stained weakly positive only in biliary ducts in the portal tract; –, tissues stained negative; ND, not determined.

### MATERIALS AND METHODS

### Animals

Three-month-old female BALB/c and CBA mice, 6-month-old female Sprague–Dawley rats (Harlan-Sprague-Dawley, Indianapolis, IN, USA), 6-month-old female New Zealand White rabbits (Hare Marland, Hewitt, NJ, USA) and two adult male baboons (*Papio cynomolgus*) raised at the Southwest Foundation for Biomedical Research (San Antonio, TX, USA) were used as sources of tissues.

### Tissues and cell lines

Table 1 lists the various tissues obtained from mice, rats, rabbits and baboons. All tissues were fixed in 10% buffered formaldehyde and embedded into paraffin blocks. The human colorectal carcinoma cell line SW1116 and the gastric carcinoma cell line Kato III were obtained from the American Type Culture Collection (Rockville, MD, USA).

### Antibodies

MAbs CO17–1A and GA733 (Herlyn et al, 1979, 1984; Gottlinger et al, 1986) were purified from ascites on protein A–agarose columns. For production of rabbit polyclonal antibodies to GA733 antigen, rabbits were immunized with the antigen purified from Nonidet P40 extracts of the human colon carcinoma cells SW1116 on immunoaffinity columns coupled with MAb GA733 (Ross et al, 1986). Rabbits were immunized subcutaneously three times with 31 μg (first injection) of antigen in Freund's complete adjuvant and 15.5 μg (second and third injections) of antigen in incomplete adjuvant. These polyclonal antibodies contain antibodies directed to the epitopes defined by MAbs GA733 and CO17–1A on the antigen, based on the demonstrated inhibition (49–65%) of binding of the MAbs defining these epitopes to colorectal carcinoma cells by the rabbit polyclonal antibodies to GA733 antigen but not by normal rabbit IgG (results not shown).

For production of rabbit polyclonal antibodies to the extracellular domain of mEGP (mEGP-EC) antigen, rabbits were immunized subcutaneously with 52 μg of recombinant antigen (for production and purification of the antigen, see below) in Freund's complete adjuvant (first injection) and 16 μg of antigen in incomplete adjuvant (second and third injections). Antibodies were isolated from immune rabbit sera on protein A–agarose columns. Normal murine and rabbit immunoglobulins (Organon Teknika, Durham, NC, USA) were used as negative controls.

All antibodies were dialysed against 0.1 M sodium bicarborate, pH 8.2–8.6, and incubated with biotin (120 μg of NHS-LC-biotin per 1 mg of antibody; Pierce Chemical, Rockford, IL, USA) in dimethylsulphoxide (DMSO) for 4 h at room temperature. The labelled antibody preparations were then dialysed against phosphate-buffered saline (PBS). Biotinylated rabbit polyclonal antibodies to GA733 antigen and MAb GA733 all bound specifically to their corresponding antigens in enzyme-linked immunosorbent assay (ELISA). However, MAb CO17–1A lost its binding reactivity to GA733 antigen after biotinylation and was not included in the immunoperoxidase assays.

### Direct immunohistoperoxidase method

Four serial sections from each tissue sample were deparaffinized, rehydrated, washed and incubated with 3% hydrogen peroxide in methanol for 15 min to block endogenous peroxidase. Sections were then washed with PBS and incubated overnight at 4°C with a 1:10 dilution of normal mouse serum (for staining with biotinylated MAb) or normal rabbit serum (for staining with biotinylated rabbit antibodies). Sections were further incubated with the various preparations of biotinylated antibodies at 100 μg $ml^{-1}$ for 1 h at room temperature, washed and incubated with avidin–peroxidase complex (ABC VectaStain Reagent Kit, Vector, Burlingame, CA, USA) for 1 h at room temperature. The peroxidase

© Cancer Research Campaign 1997

reaction was developed with diaminobenzidine (5 mg in 10 ml of PBS containing 10 µl of 30% hydrogen peroxide) for 3 min at room temperature. Sections were washed, counterstained with haematoxylin/eosin, dehydrated and mounted according to standard procedures.

### Immunoprecipitation

Single-cell suspensions were prepared from various BALB/c mouse tissues or Kato III human gastric carcinoma cells. Cells in suspension ($5 \times 10^7$ cells in 10 ml of isotonic Tris buffer, pH 7.5) were biotinylated (NHS-LC-biotin, 100 µl of 10 mg $ml^{-1}$ stock DMSO, Pierce Chemical, Rockford, IL, USA) to label cell-surface molecules. Aliquots of the cells were lysed in RIPA buffer [20 mM Tris-HCl, pH 8.5, 150 mM sodium chloride, 5 mM EDTA, 1% Triton X-100, 1% deoxycholic acid, 0.1% sodium dodecyl sulphate (SDS), 1 mM phenylmethylsulphonyl fluoride (PMSF)] and centrifuged, and the supernatants were incubated with protein A–Sepharose beads to remove non-specifically bound material. Supernatants were then incubated overnight with protein A–Sepharose beads coupled with antibody (1:40 serum dilution or 400–800 µg $ml^{-1}$ of MAb CO17–1A or GA733). After six washes with RIPA buffer, samples were boiled in 50 µl of SDS–polyacrylamide gel electrophoresis (PAGE) sample buffer for 3 min and centrifuged, and 25 µl of the supernatant was separated on a 12% SDS-PAGE and transferred to nitrocellulose. The blot was blocked [3% bovine serum albumin (BSA) in 50 mM Tris-HCl, pH 7.5, 500 mM sodium chloride, 0.1% Tween-20], incubated with alkaline phosphatase-conjugated streptavidin (1:5000, Sigma ImmunoChemicals, St Louis, MO, USA) and developed with substrate solution.

### Recombinant mEGP-EC production

Oligonucleotide primers were synthesized by automated phosphoramidite chemistry on a model 380A DNA synthesizer (Applied Biosystems, Foster City, CA, USA). mEGP-EC was amplified by polymerase chain reaction (PCR) from mEGP cDNA template (1 ng) (Bergsagel et al, 1992). A 795-bp product was generated using PCR primers containing a *Pst*I site (5′ end, bp 108) (Isberg and Leong, 1990) and a *Xba*I site and translation stop codon (3′ end, bp 902) (Sommers and Smith, 1987). The production of the transfer vector and, subsequently, of mEGP-EC was essentially the same as that described for the human GA733 antigen (Strassburg et al, 1991). Briefly, the baculovirus vector pVL1392 containing the 796-bp fragment coding for the EC of mEGP was constructed by unidirectional cloning of the mEGP-EC cDNA into the *Xba*I–*Pst*I site. Sequencing of mEGP-EC subcloned from the pVL1392 recombinant into pUC-19 confirmed identity with the published mEGP sequence (Bergsagel et al, 1992). The recombinant baculovirus vector and Baculogold viral DNA (Pharmingen, San Diego, CA, USA) were cotransfected into Sf9 insect cells (Sommers and Smith, 1987) grown in Grace's insect cell medium (Gibco, BRL, Gaithersburg, MD, USA) supplemented with 10% fetal bovine serum to obtain recombinant baculovirus mEGP-EC. Recombinant virus from culture supernatant (passage 1) was used to infect High-Five insect cells (Invitrogen Corporation, San Diego, CA, USA) to produce passage 2 virus stocks. The passage 2-infected cells were grown in serum-free medium (SF900, II; Gibco, BRL) for 72 h. A sample of the culture supernatant (containing secreted mEGP-EC) was subjected to SDS-PAGE, and the gel was stained with Coomassie blue. Secreted mEGP-EC was purified from culture supernatant on a Mono-Q column (Pharmacia, Uppsala, Sweden) following the manufacturer's procedure. Protein concentration in the purified antigen preparation was determined using the method of Lowry et al (1951).

### ELISA

Binding reactivities between recombinant proteins and antibodies were determined by ELISA (Somasundaram et al, 1995). Briefly, ELISA plates (Corning Glass Works, Corning, NY, USA) were coated with different amounts of antigen (GA733-EC, mEGP-EC or BSA) overnight at 4°C, blocked, washed and treated with various dilutions of polyclonal rabbit anti-mEGP-EC or anti-GA733-EC antibodies, MAb to GA733 antigen or control antibodies. After incubation, plates were washed and peroxidase-labelled secondary antibodies (goat anti-mouse $F(ab')_2$ or anti-rabbit IgG; Accurate Chemical and Scientific, Westbury, NY, USA) were added for 1 h at room temperature. Plates were washed and ABTS Microwell peroxidase substrate (Kirkegaard & Perry Labs, Gaithersburg, MD, USA) was added. Reactions were terminated by addition of 0.5 M sodium hydroxide, and optical density (OD) at 405 nm was determined.

### RESULTS

### Reactivities of tissues from experimental animals with polyclonal rabbit antibodies to the GA733 antigen in immunohistoperoxidase assay

Table 1 summarizes the distribution of the GA733 antigen homologue on various animal tissues as detected by polyclonal rabbit antibodies to the human antigen in the immunohistoperoxidase assay. The intestinal mucosa was stained in mice, rats and non-human primates but not in rabbits. In addition, the liver of mice, rats and non-human primates reacted weakly, but specifically, with polyclonal antibodies to the GA733 antigen, in particular in biliary ducts in the portal tract. Baboons also expressed the antigen on the distal part of the common bile duct. Apical staining of columnar cells of the mucosa was predominant in the intestine of mice, rats and baboons (Figures 1–3 respectively). In baboons, the goblet cells of the mucosa also showed clear reactivity (Figure 3), similar to the findings in human intestine (Figure 4). Secreted mucins were strongly positive in mouse, baboon and human tissues (Figures 1, 3 and 4) but weakly positive in rat tissues (Figure 2).

Despite these differences, the common feature of antigen expression in normal tissues of mice, rats, non-human primates and humans is the presence of the antigen in intestinal mucosa from duodenum to lower rectum.

### Non-reactivity of tissues of experimental animals with MAb to the GA733 antigen

None of the tissues that were derived from the various animal species and that were reactive with polyclonal antibodies to GA733 (Table 1) were positive for the epitope defined by the MAb GA733 (not shown), which was detected in human colonic tissue (Figure 5), in agreement with our previous results (Herlyn et al, 1984). In normal human colon, MAb GA733 stained only the basal parts of columnar cells, mostly in upper, differentiated parts of intestinal crypts or villi (Figure 5), whereas polyclonal antibodies also stained goblet cells and secreted mucins (Figure 4).

© Cancer Research Campaign 1997

**Figure 1** Expression of the GA733 antigen homologue in normal colon of a BALB/c mouse. (**A**) Direct immunoperoxidase staining with biotinylated polyclonal rabbit antibodies to the GA733 antigen. (**B**) Absence of staining with biotinylated normal rabbit IgG (× 160 magnification; haematoxylin/eosin counterstain)

**Figure 2** Expression of the GA733 antigen homologue in normal colon of a Sprague-Dawley rat. For **A** and **B**, see Figure 1 legend (× 160 magnification; haematoxylin/eosin counterstain)

### Immunoprecipitation of the GA733 antigen homologue from normal mouse tissues with polyclonal antibodies to mEGP-EC or GA733 antigen

Using rabbit polyclonal antibodies to mEGP-EC, a 40-kDa antigen was precipitated from mouse colon (Figure 6, lane c), normal mouse small intestine, stomach, kidney and lung tissues but not from any other normal tissues tested (Table 2). These immunoprecipitation data correlate well with results obtained previously by Northern blotting (Bergsagel et al, 1992). A 40-kDa antigen was also precipitated with rabbit polyclonal antibodies to GA733 antigen from both Kato III human gastric carcinoma cells (Figure 6, lane a) and normal mouse colon (Figure 6, lane b) and intestine but not from any other tissues tested (Table 2). These antibodies non-specifically precipitated two minor higher molecular weight contaminants from normal mouse colon (Figure 6, lanes b and d). Rabbit anti-BSA antibody precipitated neither the human GA733 antigen from Kato III cells (not shown) nor the 40-kDa GA733 antigen homologue from normal mouse intestine (Figure 6, lane d). MAbs GA733 and CO17-1A precipitated the antigen from Kato III cells but not from normal mouse colon. Furthermore, the antigen homologue could not be isolated from NP-40 extracts of normal mouse colon on immunoaffinity columns coupled with MAb GA733 or CO17-1A (not shown). The molecular mass (40 kDa) estimated for the immunoprecipitated murine antigen by SDS-PAGE is consistent with the estimated molecular mass (37.4 kDa) based on the deduced amino acid sequence of the mature protein and the assumption that each of the two potential glycosylation sites has a 5-kDa sugar moiety (Bergsagel et al, 1992).

### Immunological cross-reactivity between human GA733 antigen and mEGP-EC derived from recombinant baculovirus

To demonstrate immunological cross-reactivity between isolated human GA733 and mEGP proteins, the reactivity of recombinant baculovirus-derived GA733-EC (Strassburg et al, 1991) with polyclonal antibodies to mEGP-EC and baculovirus-derived mEGP-EC (described below) with either polyclonal or monoclonal antibodies to GA733-EC was determined.

Supernatants of High-Five cells infected with recombinant baculovirus mEGP-EC were monitored by SDS-PAGE to evaluate expression and secretion of mEGP-EC purified on an anion exchange column. Purified mEGP-EC protein migrated on SDS-PAGE with an apparent mass of about 33 kDa (Figure 7, lane c),

© Cancer Research Campaign 1997

**Figure 3** Expression of the GA733 antigen homologue in normal colon of a baboon. For **A** and **B**, see Figure 1 legend (× 160 magnification; haematoxylin/eosin counterstain)

**Figure 4** Expression of the GA733 antigen by normal human colon. For **A** and **B**, see Figure 1 legend (× 160 magnification; haematoxylin/eosin counterstain)

which is similar to the apparent size of the GA733-EC expressed in insect cells and purified by immunoaffinity chromatography on MAb GA733-coupled Sepharose (Strassburg et al, 1991; Figure 7, lane b). Both proteins are heterogeneously glycosylated, which is probably responsible for their migration as diffuse bands on SDS gels at a somewhat higher apparent molecular mass compared with the actual size.

Direct analysis of purified mEGP-EC by MALDI mass spectrometry showed substantial mass heterogeneity, with a predominant mass at about 28 496 Da. The difference between this observed mass and the predicted mass of 27 712 Da calculated from the amino acid sequence after signal peptide removal probably results from glycosylation. This mass difference and the observed heterogeneity of mEGP-EC are similar to the heterogeneity obtained for both the full-length and extracellular forms of the GA733 antigen using mass spectrometry (data not shown). The discrepancy between the actual mass of mEGP-EC of about 28.5 kDa determined by mass spectrometry and the apparent mass of about 33 kDa on SDS gels reflects anomalous protein migration on the gels, as is typically observed for glycoproteins.

Recombinant mEGP-EC was used as a target for polyclonal and monoclonal antibodies to the human GA733 antigen in ELISA. For comparison, human GA733 antigen was used as a target for polyclonal antibodies to mEGP-EC. Polyclonal rabbit antibodies, but not MAb GA733 or CO17-1A, to the human GA733 antigen showed significant and specific binding to purified recombinant mEGP-EC (Table 3). Binding of polyclonal anti-GA733 antibodies to mEGP-EC was lower than that of polyclonal anti-mEGP-EC antibodies to mEGP-EC. Polyclonal anti-mEGP-EC antibodies also bound significantly to the human antigen, although at lower levels than the antibodies elicited by the human antigen (Table 3).

### DISCUSSION

The human colorectal carcinoma-associated antigen GA733 expressed on both tumour and normal tissues is one of the best studied targets for experimental and clinical cancer immunotherapy. However, the relevance of preclinical studies of targeting this antigen in passive and active immunotherapy (with MAbs, anti-idiotypic antibodies, and recombinant antigen; reviewed in Herlyn et al, 1982, 1995, 1996) for clinical trials in patients has been difficult to determine in the absence of information on normal tissue expression of the antigen homologue in animals. We demonstrate here that an antigen homologous to the human gastrointestinal carcinoma-associated antigen GA733 is expressed by normal intestine and liver of mice, rats and non-human primates, as determined by immunohistoperoxidase staining of tissues. The common feature of GA733 antigen homologue

**Figure 5** Expression of the GA733 epitope in human colon tissue. Tissue was stained with biotinylated MAb GA733 (**A**) or normal mouse immunoglobulin (**B**) in direct immunoperoxidase assay (× 160 magnification; haematoxylin/eosin counterstain)

**Figure 6** Immunoprecipitation of the GA733 antigen from human gastric carcinoma cells Kato III (lane a) and of the antigen homologue from normal murine colon tissue (lanes b and c). Equivalents of 6.25 × 10$^6$ cells were loaded per lane. Antibodies: lanes a and b, rabbit sera to the GA733 antigen; lane c, rabbit sera to mEGP; lane d, rabbit sera to BSA. Nitrocellulose immunoblot was developed with alkaline phosphatase-labelled streptavidin and substrate. Molecular size ($M_r$) markers were run in an adjacent lane

**Table 2** Detection of mEGP antigen on mouse tissues (BALB/c) by immunoprecipitation and Northern blot analysis

| | Immunoprecipitation[a] | | |
|---|---|---|---|
| **Tissue** | **Anti-mEGP-EC antibody** | **Anti-GA733 antibody** | **Northern blotting[b]** |
| Stomach | + | – | + |
| Small intestine | + | + | + |
| Colon | + | + | + |
| Kidney | + | – | + |
| Lung | + | – | + |
| Heart | – | – | – |
| Muscle | – | – | – |
| Liver | – | – | – |
| Brain | – | – | – |
| Spleen | – | – | – |

[a]Immunoprecipitation of mEGP with rabbit anti-mEGP-EC or anti-GA733 antigen sera was performed as described in Materials and methods. Control antibodies (rabbit anti-BSA) showed no reactivity. [b]Bergsagel et al (1992).

**Figure 7** SDS-PAGE of recombinant GA733-EC and mEGP-EC. Purified recombinant proteins were subjected to SDS-PAGE on a 10% gel in the absence of reducing reagents, and the gel was stained with Coomassie blue. Molecular size markers were run in the right lane. (a) Full-length recombinant GA733 antigen (8 μg), (b) GA733-EC (4 μg) and (c) mEGP-EC (4 μg). The full-length GA733 recombinant protein showed a major monomer band and a minor dimer band expression in normal animal tissues is its presence in intestinal secretions and mucosa from duodenum to lower rectum. Staining of additional cell types of intestine with polyclonal antibodies to human GA733 antigen varied somewhat for the different species and may reflect species differences in the cellular distribution of various antigenic epitopes of the antigen homologue. Alternatively, more than one gene may regulate expression of the various epitopes recognized by the polyclonal antibodies.

SDS-PAGE analyses with polyclonal antibodies to mEGP-EC revealed the expression of the antigen homologue in mouse intestine,

 © Cancer Research Campaign 1997

**Table 3** Immunological cross-reactivity between the human GA733-EC and mEGP-EC

| Antibody[a] | Antibody binding (OD at 405 nm) | |
|---|---|---|
| | mEGP-EC | GA733-EC |
| Rabbit αGA733-EC antigen | 0.301[b] | 0.438[b] |
| Rabbit αmEGP-EC | 0.471[b] | 0.299[b] |
| MAb GA733 | 0.016 | 0.390[b] |
| MAb CO17-1A | 0 | 0.240[b] |
| Normal rabbit IgG | 0.028 | 0.034 |
| Normal mouse IgG | 0.016 | 0.039 |

[a]Antibodies were used at optimal concentrations (2–10 μg $ml^{-1}$) showing maximal binding values in ELISA. [b]Values are significantly higher ($P < 0.05$; Student's *t*-test) than control values (obtained with normal IgG on specific target or immune IgG on BSA target).

stomach, lung and kidney tissues. These tissues express mEGP RNA (Bergsagel et al, 1992). However, the antigen was not detected on these tissues (with the exception of intestine) by immunoprecipitation or by immunohistoperoxidase staining with polyclonal antibodies to human GA733. Thus, the murine antigen homologue can be detected in normal mouse intestine by antibodies directed to both the murine and human antigen, whereas its detection in normal mouse stomach, lung and kidney tissues is restricted to antibodies directed to the murine antigen. These differences most likely reflect the greater sensitivity of antigen detection using the homologous compared with the heterologous antibody. Therefore, it is possible that in rats and baboons, similar to mice, tissues in addition to intestine and liver found positive in immunohistoperoxidase assay with antibodies to the human GA733 antigen express the antigen homologue; although it is difficult to test this hypothesis as antibodies to the rat and baboon antigen homologues are not available.

mEGP and the rat and baboon antigen homologues might be expressed at low levels in the liver, in particular in biliary ducts in the portal tract, as determined in immunohistoperoxidase assay; but these results could not be confirmed by immunoprecipitation of mouse liver tissues with antibodies to mEGP-EC, in addition mEGP mRNA is absent in these tissues (Bergsagel et al, 1992). The expression of the antigen in a small subset of liver cells may render its detection by immunoprecipitation and Northern blotting difficult.

Expression of the GA733 antigen homologue mEGP in normal mouse tissues is consistent with the previous description of a murine cDNA with ~80% sequence homology (in the coding region) to the human GA733 cDNA (Bergsagel et al, 1992); however, their study did not investigate the expression of the antigen by normal mouse tissues. Importantly, we have provided direct evidence for the immunological cross-reactivity between mEGP and the human GA733 antigen. Thus, polyclonal antibodies to the human antigen bound specifically to murine intestinal tissue. Furthermore, recombinant mEGP-EC protein bound to antibodies elicited by the human GA733 antigen, and GA733 antigen bound to antibodies elicited by mEGP-EC.

In mice, the CO17-1A epitope was absent in immunoprecipitation and immunoaffinity antigen isolation analyses of normal intestinal tissue. Furthermore, in mice, rats and non-human primates, immunohistoperoxidase staining of normal tissues did not detect the epitope defined by MAb GA733, although this epitope is expressed by human tissues (Herlyn et al, 1984). In contrast, Shetye et al (1990) have reported the staining of normal colonic and pancreatic tissues with MAb GA733 in rats. This discrepancy may be a result of the different detection methods used, i.e. direct immunohistoperoxidase assay with purified MAb in the present study vs indirect assay with MAb in tissue culture supernatant in the study by Shetye et al (1990). We used the direct assay using biotinylated MAb to the GA733 antigen because of its high specificity. The indirect assay, in which we used sequentially unlabelled, purified MAb and labelled anti-mouse immunoglobulin antibody, did not reveal specific binding of MAb GA733 to rat tissues because of high non-specific binding of control mouse immunoglobulin (results not shown).

The absence of the GA733 and CO17-1A epitopes on normal mouse tissues is consistent with the demonstrated absence of these epitopes on baculovirus-derived mEGP-EC. Furthermore, the GA733 epitope is absent on murine plasmacytoma cells, despite the presence of mEGP mRNA in these cells (Bergsagel et al, 1992). Given that the GA733 and CO17-1A epitopes are absent on animal tissues, we speculate that in humans these epitopes are encoded by those sequences in the human GA733 antigen (residues 3–45 or 150–186) that differ from the corresponding sequences of the murine antigen homologue.

Thus, the antigen identified in animal tissues by the rabbit polyclonal antibodies is similar, but not identical, to the human GA733 antigen, and both antigens show similar tissue distribution (Herlyn et al, 1984; Gottlinger et al, 1986; Shetye et al, 1988).

The non-reactivity of rabbit tissues with rabbit polyclonal antibodies to the human GA733 antigen might reflect the presence of antibodies in the polyclonal preparation directed preferentially to epitopes that are absent on normal rabbit tissues. Thus, rabbits might be immunologically tolerant of those epitopes on the human antigen that are also expressed on normal rabbit tissues. Alternatively, rabbits might not express any GA733 antigen-related epitopes on their normal tissues, although this seems unlikely in light of the wide distribution of the antigen homologue in other animal species.

We have shown that mice, rats and non-human primates express the GA733 antigen homologue that lacks the CO17-1A and GA733 epitopes on some of their normal tissues. Previous studies of experimental passive and active immunotherapy targeting the human GA733 antigen in mice (Herlyn et al, 1982, 1984, 1995, 1997) must be interpreted with caution, keeping in mind the immunological differences between the human antigen and its homologue. Thus, passive immunotherapy with MAb CO17-1A and GA733 (Herlyn et al, 1982, 1984) and active immunotherapy with anti-idiotypic antibodies mimicking the GA733 or CO17-1A epitope or with recombinant GA733 antigen (reviewed in Herlyn et al, 1995, 1996) have been performed in mice which, in contrast to humans, do not express the CO17-1A and GA733 epitopes on normal tissues. The differences in the GA733 epitope tissue expression between mice and humans might explain why treatment of mice with MAb GA733 was not accompanied by toxicity (Herlyn et al, 1984), whereas this MAb showed dose-limiting toxicity to gastrointestinal organs in colon cancer patients (Herlyn et al, 1991). Recently, MAb G8.8 against mEGP has been described (Farr et al, 1991; Borkowski et al, 1996). The MAb binds to various murine epithelial tissues (Farr et al, 1991), and thus it provides a valuable tool for experimental passive and active immunotherapy against mEGP using the MAb and derived anti-idiotypic antibodies respectively. Furthermore, our initial studies of active immunotherapy in mice, using recombinant human

GA733 antigen derived from baculovirus or expressed in vaccinia or adenovirus (Herlyn et al, 1995), must be followed by similar studies using recombinant mEGP vaccines. We are currently developing a murine model of active immunotherapy against mEGP that includes the well-characterized murine colon carcinoma cell line CT26 (Brattain et al, 1980). This model closely mimics the conditions in patients and thus may be predictive for future clinical trials of active immunotherapy targeting the GA733 antigen.

### ACKNOWLEDGEMENTS

This work was supported in part by National Institutes of Health Grants CA 605595, CA 10815 and CA 43735, and a grant from Ajinomoto, Japan. We thank Dr Michael Kuehl (NCI, Bethesda, MD, USA) for helpful discussions; Elizabeth Freeman and Dr John Daly (Hospital of the University of Pennsylvania, Philadelphia, PA, USA) for providing human colonic tissue; Marie Prewett, Dawn Marchadier, Elsa Aglow and Margaret Enverso for excellent technical assistance; Joyce Macauley for typing; and Marina Hoffman for editing this manuscript.

### REFERENCES

Bergsagel PL, Victor-Kobrin C, Timblin CR, Trepel J and Kuehl WM (1992) A murine cDNA encodes pan-epithelial glycoprotein that is also expressed on plasma cells. *J Immunol* **148**: 590–596

Borkowski TA, Nelson AJ, Farr AG and Udey MC (1996) Expression of gp40, the murine homologue of human epithelial cell adhesion molecule (Ep-CAM), by murine dendritic cells. *Eur J Immunol* **26**: 110–114

Brattain MG, Strobel-Stevens J, Fine D, Webb M and Sarrif AM (1980) Establishment of mouse colonic carcinoma cell lines with different metastatic properties. *Cancer Res* **40**: 2142–2146

Farr A, Nelson A, Truex J and Hosier S (1991) Epithelial heterogeneity in the murine thymus: a cell surface glycoprotein expressed by subcapsular and medullary epithelium. *J Histochem Cytochem* **39**: 645–653

Frodin J-E, Harmenberg U, Biberfeld P, Christensson B, Lefvert A-K, Reiger A, Shetye J, Warren B and Mellstedt H (1988) Clinical effects of monoclonal antibodies (MAB 17-1A) in patients with metastatic colorectal carcinomas. *Hybridoma* **7**: 309–321

Gottlinger HG, Funke I, Johnson JP, Gokel JM and Riethmuller G (1986) The epithelial cell surface antigen 17-1A, a target for antibody-mediated tumor therapy: its biochemical nature, tissue distribution and recognition by different monoclonal antibodies. *Int J Cancer* **38**: 47–53

Hamby CV, Liao SK, Kanamaru T and Ferrone S (1987) Immunogenicity of human melanoma-associated antigens defined by murine monoclonal antibodies in allogeneic and xenogeneic hosts. *Cancer Res* **47**: 5284–5289

Herlyn D and Koprowski H (1982) IgG2a monoclonal antibodies inhibit human tumor growth through interaction with effector cells. *Proc Natl Acad Sci USA* **79**: 4761–4765

Herlyn M, Steplewski Z, Herlyn D and Koprowski H (1979) Colorectal carcinoma-specific antigen: detection by means of monoclonal antibodies. *Proc Natl Acad Sci USA* **76**: 1438–1442

Herlyn D, Herlyn M, Ross AH, Ernst C, Atkinson B and Koprowski H (1984) Efficient selection of human tumor growth-inhibiting monoclonal antibodies. *J Immunol Meth* **73**: 157–167

Herlyn D, Wettendorff M, Schmoll E, Iliopoulos D, Schedel I, Dreikhausen U, Raab R, Ross AH, Jaksche H, Scribe M and Koprowski H (1987) Anti-idiotype immunization of cancer patients: modulation of the immune response. *Proc Natl Acad Sci USA* **84**: 8055–8059

Herlyn D, Sears HF, Ernst CS, Iliopoulos D, Steplewski Z and Koprowski H (1991) Initial clinical evaluation of two murine IgG2a monoclonal antibodies for immunotherapy of gastrointestinal carcinoma. *Am J Clin Oncol* **14**: 371–378

Herlyn D, Harris D, Zaloudik J, Sperlagh M, Maruyama H, Jacob L, Kieny M-P, Scheck S, Somasundaram R, Hart E, Ertl H and Mastrangelo MJ (1994) Immunomodulatory activity of monoclonal anti-idiotypic antibody to anti-colorectal carcinoma antibody CO17-1A in animals and patients. *J Immunother* **15**: 303–311

Herlyn D, Somasundaram R, Zaloudik J, Li W, Jacob L, Harris D, Kieny M-P, Ricciardi R, Gonczol E, Sears H and Mastrangelo M (1995) Cloned antigens and antiidiotypes. *Hybridoma* **14**: 159–166

Herlyn D, Somasundaram R, Li W and Maruyama H (1996) Anti-idiotype cancer vaccines–past and future. *Cancer Immunol Immunother* **43**: 65–76

Herlyn D, Somasundaram R, Jacob L, Li W, Zaloudik J, Maruyama H, Benden A, Harris D and Mastrangelo M (1997) Anti-idiotypic antibodies that mimic the colorectal cancer antigen CO17-1A/GA733: twelve years of preclinical and clinical studies. In *Idiotypes in Medicine, Infections Autoimmunity and Cancer*, Shoenfeld M, Kennedy R and Ferrone S. (eds), Elsevier Pergamon: Holland (in press)

Isberg RR and Leong JM (1990) Multiple $\beta_1$ chain integrins are receptors for invasin, a protein that promotes bacterial penetration into mammalian cells. *Cell* **60**: 861–871

LoBuglio AF, Saleh MN, Lee J, Khazaeli MB, Carrano R, Holden H and Wheeler RH (1988) Phase I trial of multiple large doses of murine monoclonal antibody CO17-1A. I. Clinical aspects. *J Natl Cancer Inst* **80**: 932–936

Lowry OH, Rosebrough NJ, Farr AL and Randall RJ (1951) Protein measurement with the Folin phenol reagent. *J Biol Chem* **193**: 265–275

Mach J-P, Chatal J-F, Lumbroso J-D, Buchegger F, Forni M, Ritschard J, Berche C, Douillard JY, Carrel S, Herlyn M, Steplewski Z and Koprowski H (1983) Tumor localization in patients by radiolabeled monoclonal antibodies against colon carcinoma. *Cancer Res* **43**: 5593–5600

Perez MS and Walker LE (1989) Isolation and characterization of a cDNA encoding the KS1/4 epithelial carcinoma marker. *J Immunol* **142**: 3662–3667

Riethmüller G, Schneider-Gädicke E, Schlimok G, Schmiegel W, Raab R, Höffken K, Gruber R, Pichlmaier H, Hirche H, Pichlmaier R, Buggisch P and Witte J. The German Cancer Aid 17-1a Study Group (1994) Randomised trial of monoclonal antibody for adjuvant therapy of resected Dukes' C colorectal carcinoma. *Lancet* **343**: 1177–1183

Ross AH, Herlyn D, Iliopoulos D and Koprowski H (1986) Isolation and characterization of a carcinoma-associated antigen. *Biochem Biophys Res Commun* **135**: 297–303

Sears HF, Herlyn DM, Herlyn M, Grotzinger PJ, Steplewski Z and Koprowski H (1981) *Ex vivo* perfusion of a tumor-containing colon with monoclonal antibody. *J Surg Res* **31**: 145–150

Sears HF, Herlyn D, Steplewski Z and Koprowski H (1984) Effects of monoclonal antibody immunotherapy on patients with gastrointestinal adenocarcinoma. *J Biol Resp Mod* **3**: 138–150

Shetye J, Frodin J-E, Christensson B, Grant C, Jacobsson B, Sundelius S, Mikael S, Biberfeld P and Mellstedt H (1988) Immunohistochemical monitoring of metastatic colorectal carcinoma in patients treated with monoclonal antibodies (MAB 17-1A). *Cancer Immunol Immunother* **27**: 154–162

Shetye JD, Rubio CA, Harmenberg U, Ware J, Duvander A and Mellstedt HT (1990) Tumor-associated antigens common to humans and chemically-induced colonic tumors of the rat. *Cancer Res* **50**: 6358–6363

Simon B, Podolsky DK, Moldenhauer G, Isselbacher KJ, Gattoni-Celli S and Brand SJ (1990) Epithelial glycoprotein is a member of a family of epithelial cell surface antigens homologous to nidogen, a matrix adhesion protein. *Proc Natl Acad Sci USA* **87**: 2755–2759

Somasundaram R, Zaloudik J, Jacob L, Benden A, Sperlagh M, Hart E, Marks G, Kane M, Mastrangelo M and Herlyn D (1995) Induction of antigen-specific T and B cell immunity in colon carcinoma patients by anti-idiotypic antibody. *J Immunol* **155**: 3253–3261

Sommers MD and Smith GE (1987) A manual of methods for baculovirus vectors and insect cell culture procedures. Agricultural Experiment Station Bulletin No. 1555. *Tex Agric Exp St Bull* **1555**: 5–56

Strassburg CP, Kasai Y, Seng BA, Zaloudik J, Herlyn D, Koprowski H and Linnenbach AJ (1991) Baculovirus recombinant expressing a secreted form of a trans-membrane carcinoma-associated antigen. *Cancer Res* **52**: 815–821

Strnad J, Hamilton AE, Beavers LS, Gamboa GC, Apelgren LD, Taber LD, Sportsman JR, Bumol TF, Sharp JD and Gadski RA (1989) Molecular cloning and characterization of a human adenocarcinoma/epithelial cell surface antigen complementary DNA. *Cancer Res* **49**: 314–317

Szala S, Froehlich M, Scollon M, Kasai Y, Steplewski Z, Koprowski H and Linnenbach AJ (1990) Molecular cloning of cDNA for the carcinoma-associated antigen GA733-2. *Proc Natl Acad Sci USA* **87**: 3542–3546

Wettendorff M, Iliopoulos D, Tempero M, Kay D, Defreitas E, Koprowski H and Herlyn D (1989) Idiotypic cascades in cancer patients treated with monoclonal antibody CO17-1A. *Proc Natl Acad Sci USA* **86**: 3787–3791

© Cancer Research Campaign 1997